# Fine-Scale Habitat Associations of a Terrestrial Salamander: The Role of Environmental Gradients and Implications for Population Dynamics

**DOI:** 10.1371/journal.pone.0062184

**Published:** 2013-05-06

**Authors:** William E. Peterman, Raymond D. Semlitsch

**Affiliations:** University of Missouri, Division of Biological Sciences, Columbia, Missouri, United States of America; Universität Zurich, Switzerland

## Abstract

Environmental gradients are instrumental in shaping the distribution and local abundance of species because at the most fundamental level, an organism’s performance is constrained by the environment it inhabits. In topographically complex landscapes, slope, aspect, and vegetative cover interact to affect solar exposure, creating temperature-moisture gradients and unique microclimates. The significance of the interaction of abiotic gradients and biotic factors such as competition, movement, or physiology has long been recognized, but the scale at which these factors vary on the landscape has generally precluded their inclusion in spatial abundance models. We used fine-scale spatial data relating to surface-soil moisture, temperature, and canopy cover to describe the spatial distribution of abundance of a terrestrial salamander, *Plethodon albagula*, across the landscape. Abundance was greatest in dense-canopy ravine habitats with high moisture and low solar exposure, resulting in a patchy distribution of abundance. We hypothesize that these patterns reflect the physiological constraints of Plethodontid salamanders. Furthermore, demographic cohorts were not uniformly distributed among occupied plots on the landscape. The probability of gravid female occurrence was nearly uniform among occupied plots, but juveniles were much more likely to occur on plots with lower surface temperatures. The disconnect between reproductive effort and recruitment suggests that survival differs across the landscape and that local population dynamics vary spatially. Our study demonstrates a connection between abundance, fine-scale environmental gradients, and population dynamics, providing a foundation for future research concerning movement, population connectivity, and physiology.

## Introduction

Gradients of biotic and abiotic factors are inherent to heterogeneous landscapes [1]. Abiotic factors such as temperature, water, sunlight, pH, and nutrient concentrations, and biotic factors such as competition, prey availability, and predators, interact to determine species distributions and local population abundance [2]. Temperature is a critical abiotic factor related to species distribution at a regional scale, but temperature also can vary across small spatial scales. Climate and elevation generally dictate the temperature of a region [3], [4], but topographic and vegetative characteristics of the landscape affect temperature locally [5], [6]. Topography and slope influence the amount of solar exposure and hence surface temperatures [4], [7], which in turn can have significant effects on soil moisture [8]. The land cover overlaying the physical landscape (e.g. forest, grassland) further influences the amount of solar exposure to create microclimates with unique temperature-moisture characteristics [5], [9].

The spatial distributions of both plants and animals are often closely tied to microclimatic conditions [9], emphasizing the importance of understanding the environment as a proximate cause of patterns in species distribution and abundance. Gradients created by slope and aspect play a significant role in structuring plant communities [10], [11], [12] and in determining the distribution and abundance of animals ranging from carabid beetles [13] and butterflies [14], to amphibians [15] and birds [16].

Acquisition and assimilation of energy from the environment is critical for survival and reproduction, and the distribution of temperature, water, and food resources can shape the life history, abundance, and distribution of species [17]. In choosing optimal thermal environments, an organism seeks to maximize its energy consumption while minimizing metabolic expenditures [18], and in some species, rates of evaporative water loss are closely tied to temperature [19]. Different taxa perceive and relate to their environment at different scales, with many terrestrial animals operating on a scale of meters or less. Vagile animals such as insects, birds, and reptiles often exhibit differential success in relation to microclimate, and actively select favorable microclimatic conditions [14], [20], [21]. Although plants cannot actively select their local environment, seedling recruitment can nonetheless be affected by environmental gradients [22], thus shaping spatial patterns of distribution and abundance [23]. Many animals are much more plant-like in their habitat associations. While extremely dependent upon suitable temperature and moisture microclimates, terrestrial gastropods have limited dispersal abilities [24], [25]. Similarly, terrestrial salamanders of the family Plethodontidae are generally highly philopatric [26], exhibiting minimal dispersal [27]. The physical and behavioral constraints of dispersal-limited taxa make such species much more reliant on their immediate surroundings, limiting active selection of a favorable microclimate.

Plethodontid salamanders are unique among terrestrial vertebrates in that they are lungless; respiration occurs predominantly across the skin surface. Water balance is critical for survival in plethodontid salamanders, and has been shown to be an important determinant of surface activity [15], [28]. Temperature and moisture conditions must be suitable for an adequate duration of time to allow salamanders to successfully forage and meet their energy requirements [29]. From this energy budget perspective [30], a species’ distribution will be limited to areas with a positive energy budget, and local abundance may positively correlate with energy budget surplus [31].

One of the primary goals of ecology is to understand how biotic and abiotic factors influence species’ distribution and abundance [17]. These foundational relationships between an organism and its environment are often the basis of subsequent ecological inquiry into local population dynamics [32], dispersal [33], and evolutionary potential [34]. More than ever, research concerning species habitat relationships is concerned with potential responses to, or outcomes of anthropogenic land use and climate change [35]. One of the major limitations to accurately describing distribution or abundance is that neither can be observed perfectly [36], [37], [38]. Failure to account for observation error will result in an underrepresentation of true distribution or abundance [39] and bias estimates of covariate relationships [40]. Observation error can be accounted for using models that allow for the simultaneous estimation of species abundance/occupancy and detection probability [40], [41], [42].

We used binomial mixture models and a metapopulation sampling design [42], [43] to account for variable detection and obtain unbiased abundance estimates of a terrestrial plethodondid salamander, *Plethodon albagula*. Our objectives were to (1) determine the fine-scale environmental gradients that correlate with abundance, (2) describe the distribution of abundance across the landscape, and (3) determine the effects that environmental gradients have on population dynamics. We hypothesized that abundance in *P. albagula* would be positively associated with metrics relating to high moisture and cool temperatures and, correspondingly, would not be uniformly distributed across the landscape. Instead, we predicted that abundance would be topography-dependent with the greatest abundance in ravine habitats and lowest abundance in ridge habitats, which correspond to cool-moist and hot-dry microclimates, respectively [11]. We also hypothesized that reproductive success would be greatest in areas of high abundance, indicating favorable microclimates for survival, growth, and reproduction.

## Materials and Methods

### Ethics Statement

This research was done in compliance with all laws and regulations of the state of Missouri and the USA, and was conducted under Missouri Wildlife Collector’s Permit #15203 animal care protocol #7403 approved by the University of Missouri Animal Care and Use Committee.

### Study Site

Our study took place in east-central Missouri within the River Hills Ecoregion [44]. This physiographic region borders the Missouri River, and is characterized by forested ridges and valleys with slopes that are frequently covered by exposed rock or rock outcrops. Seasonal temperatures range from −6.8–31.2°C, and average annual rainfall is 94.2 cm. Our field site was located at Daniel Boone Conservation Area (DBCA; 38.78° N, 91.39° W; 157–280 m a.s.l.), which encompasses 1424.5 ha of mature (80–100 yrs old) second-growth forest consisting of oak (*Quercus* spp.) and hickory (*Carya* spp.) dominated overstory with varying amounts of sugar maple (*Acer saccharum*) and red cedar (*Juniperus virginiana*) in the understory [45].

### Study Species

*Plethodon albagula* (western slimy salamander) is a large plethodontid salamander that lives in forested habitats throughout the Ozark and Ouchitae mountains of Missouri, Arkansas, eastern Oklahoma, and northeastern Texas, USA. Within these forested habitats, salamanders are most frequently associated with moist, sheltered ravine and valley habitats. Like other plethodontid salamanders, *P. albagula* spend much of the year in subterranean refugia, but are surface active in the spring and autumn months when temperature and moisture conditions are favorable [46]. Foraging, dispersal, and courtship activities are generally nocturnal, and individuals retreat underground or seek refuge under rocks and logs during the day. Females lay 10–20 eggs under rotting logs, rocks, or in subterranean refugia [47]. Age at sexual maturity is unknown for *P. albagula*, but ranges from 3–5 yrs in other large plethodontid salamanders [48], [49]. Dispersal and home ranges are also largely unknown. Plethodontid slamanders are generally philopatric [47], [50], and limited data on closely related species in the *P. glutinosus* complex suggests that home ranges of adult and juvenile salamanders are small (<4.0 m^2^) [51], and do not differ significantly between sexes or age classes. Although sympatric with other terrestrial salamanders in parts of its range, *P. albagula* is the only plethodontid species present in our study region [52].

### Field Surveys

**Figure 1:**
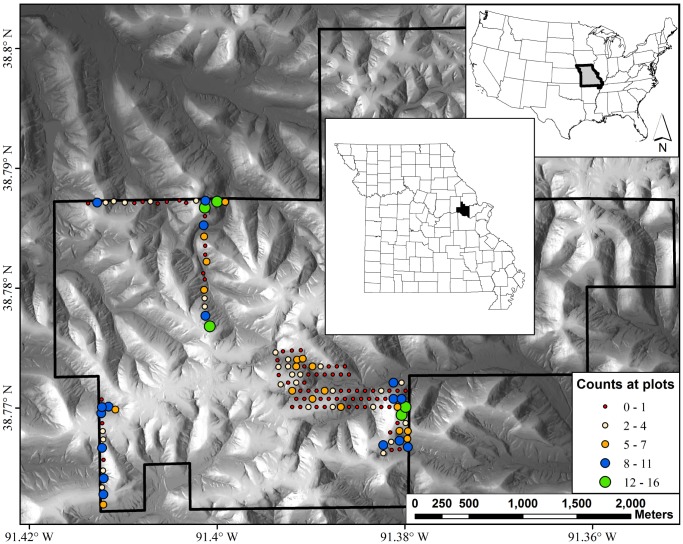
Hillshade relief map of Daniel Boone Conservation Area, MO, U.S.A. Points on the map indicate the 135 sample plot locations that are separated by at least 75 m. The counts represent the sum of all salamanders observed across the seven survey periods with no correction for imperfect detection or accounting for duplicate observations.

We surveyed for *P. albagula* using area-constrained daytime observations of 135 survey plots. Plots were 3 m×3 m, and were a minimum of 75 m apart. Due to logistical constraints, plots were not randomly selected across the landscape, but were instead arranged in an offset grid or in linear transects (Fig. 1). The location of each plot was marked in the field using a hand held GPS (Garmin 62sc) with multiple locations being taken until the estimated precision was ≤3 m. Each plot was surveyed seven times from 8 April to 28 May 2011 from 0600–1700 CST, with at least six days between surveys. During each survey, all moveable cover objects, including rocks, logs, and bark, were carefully lifted and all salamanders were captured by hand. We measured snout-vent length (SVL) and total length of each captured salamander and determined sex based on SVL and presence of a mental gland (males) [47]. If salamanders were of adult size and not visibly male (≥55 mm SVL, no mental gland), we candled the salamander to determine if eggs were present [53]. Mass was recorded to 0.01 g using a portable digital balance (Durascale, My Weigh). Cover objects were returned to their original position and salamanders were released following data collection. Leaf litter was not surveyed for salamanders because preliminary surveys deemed this largely ineffectual and too destructive of the plot habitat.

Several plot-level covariates were measured in the field. Surface soil temperature under each searched cover object was measured at a distance of 1 m during each survey using an infrared thermometer (Raytek MT4), and all measures from a plot were averaged for each survey. We also quantified the amount of available searchable cover as the surface area of logs, rocks, bark, as well as the total surface area of all these cover objects. Lastly, we measured leaf litter depth as the average of five measurements within each plot.

### Spatial Covariates

Spatial covariates were calculated in ArcGIS 9.3 (ESRI, Redlands, CA, USA). Using 1/9 arc second Nation Elevation Dataset (3 m resolution; http://seamless.usgs.gov/products/9arc.php), we derived the following spatial layers: northness (cosine of aspect; value range from 1 = north to −1 = south), eastness (sin of aspect; value range from 1 = east, −1 = west [54], slope, topographic position index (TPI), topographic wetness index (TWI), potential relative radiation (PRR), surface curvature, distance to stream, and maximum surface temperature. TPI was calculated as the slope position relative to the surrounding 90 m, with negative values indicating areas that are lower than the surrounding landscape (ravines) and positive values indicating areas that are higher than the surrounding landscape (ridges) [55]. TWI was calculated accounting for solar insolation (azimuth = 178.3, altitude = 65.3) [56]. PRR was calculated following the approach of Pierce et al. [57] wherein we estimated the relative amount of solar exposure on the landscape for every hour of the first and 15^th^ day of the months of April–October. The estimated solar exposure values for all hours and days were then summed together. Maximum surface temperature was calculated from a network of 61 Thermochron iButton data loggers (Maxim) using a hierarchical mixed-effects model as described by Fridley [4]. For each of these derived layers, we then calculated a local average of each pixel by averaging the surrounding 9 m×9 m area. In doing so, we smoothed each landscape surface as well as generalized the local landscape to help account for potential spatial error in plot location that can result from GPS imprecision. We also estimated canopy cover at our site using the normalized difference vegetation index (NDVI), which was calculated from Landsat 7 satellite imagery [58]. We obtained cloud free images of our study area for 15 June, 20 July, 9 August 2011 (http://glovis.usgs.gov/); a mean NDVI was calculated by averaging these together. Because the resolution of the NDVI layer was 30 m, we resampled it to 3 m to match the resolution of our other spatial layers.

### Temporal Covariates

Because salamander surface activity can be highly sensitive to climatological variation, we collected data on millimeters of precipitation in the 24 hrs and 5 days preceding each survey, average temperature 24 hrs preceding each survey, the number of days since a soaking rainfall event of ≥5 mm, and the number of days since any rainfall. These data were collected and averaged from three weather stations ≤20 km from our study site (http://www.wunderground.com). The Julian date of each survey was also included as a temporal covariate.

### Statistical Analyses

We analyzed the repeated observations of *P. albagula* counts at plots using binomial mixture models (i.e. N-mixture model) [42]. One of the major assumptions of binomial mixture models is that populations are closed to immigration, emigration, births, and deaths during the period of sampling. Plethodontid salamanders do exhibit vertical migration from the surface to underground refugia [46], but as long as this temporary emigration is random, then closed-population models such as the binomial mixture model should yield unbiased abundance estimates [59]. By confining our sampling to the relatively short spring active season, we feel that the closure assumptions are largely met in our system and that vertical migration (i.e. temporary emigration), which will affect detection, is dependent upon temporal climate variation. We have attempted to account for this temporal variation in detection by fitting climate covariates to our detection model. Binomial mixture models are superior to logistic or Poisson regression when modeling distribution or abundance because of their hierarchical nature. Such models allow for the estimation of species abundance as a function of site-level covariates, while accounting for imperfect species detection [37]. By correcting for variable detection of salamanders among sites and surveys, we reduce the bias in our abundance estimate to more accurately describe abundance across the landscape as it relates to environmental gradients [42], [60]. Binomial mixture models are a form of a hierarchical generalized linear mixed model [61], which can be solved through maximum likelihood estimation or Bayesian methods [39].

We analyzed our models in a Bayesian hierarchical framework using the WinBUGS software package (v. 1.4.3) [62], executed through R (v. 2.15) [63] using the R library R2WinBUGS [64]. Prior to modeling, all covariates were standardized by subtracting the arithmetic mean and dividing the standard deviation. We used uninformative normally distributed priors with a mean of zero and a variance of 10^6^ for all model parameters. Posterior summaries were based on 500,000 Markov chain Monte Carlo iterations thinned at a rate of 50 following a burn-in of 250,000 iterations. From each model we calculated the mean and 95% credible interval (CRI) for all model parameters, as well as the latent abundance parameter at each site. Model convergence was assessed using the Gelman-Rubin statistic (Rhat) [65]. Posterior predictive assessment of model fit was done using Bayesian p-value as well as a Chi-square discrepancy measure [61], [66].

We constructed our models in a five step process. (1) We fit a full model with all potential explanatory covariates in both the abundance and detection hierarchies (see Table S1), and then assessed model fit. Poisson, zero-inflated Poisson, and random effects models were all fit to our data [67]. For the random effects parameterization, a normally distributed random effects term was included in our detection model to account for unexplained variation in our plot-survey detection probability [68], [69]. (2) Using the best-fit model from step 1, we fit all covariates that could potentially affect detection of salamanders while holding abundance among sites constant. Parameter estimates for each covariate were assessed, and those that did not include zero in their 95% credible interval were retained. (3) Step 2 was then repeated with the retained covariates to confirm that the magnitude and sign of the parameter estimates did not change. (4) Using the detection model from step 3, we fit all potentially meaningful covariates to the abundance model. Parameter estimates for each covariate were assessed, and those that did not include zero in their 95% credible interval were retained. (5) Finally, the full model with all retained covariates was run and the sign and magnitude of the parameter estimates were assessed. To evaluate the predictive power of our model we conducted a leave-one-out cross-validation test. We iteratively omitted the observation data for a single site, and then re-ran the model to obtain posterior predictions for abundance at that site. These predictions were then compared to the predictions made with the full model.

Our primary model described abundance of all *P. albagula* across the landscape in relation to environmental gradients, but given this distribution, we also wanted to know if the probabilities of gravid female and juvenile salamander occurrence were the same at occupied sites. We chose these demographic groups because they represent reproductive effort (gravid females) and successful recruitment (juveniles). To address this question, we constructed multistate models using a conditional binomial parameterization in program PRESENCE v3.1 [38], [70]. Multistate models were fit separately for gravid females and for juvenile salamanders. Three states were present in each of these models: (1) no salamanders present; (2) salamanders present, but target salamander absent; (3) target salamander present, where target salamander is either gravid female or juvenile salamander, for each respective model. To parameterize the multistate model, we fit the same covariates to the detection and occupancy parameters in the multistate model as were found to be significant covariates of detection and abundance in the binomial mixture model (Table 1). The conditional state probability parameter (R), was also fit with the five significant abundance covariates.

**Table 1:** Parameter estimates for detection and abundance models in hierarchical binomial mixture model.

			95% Credible Interval	
Model	Parameter	Beta	Lower	Upper
**Detection**				
	Intercept	−1.64	−1.994	−1.293
	Date	0.904	0.694	1.139
	Bark	0.263	0.095	0.431
	Soak.Rain	−0.491	−0.724	−0.276
	Soak.Rain^2^	0.463	0.265	0.675
	Temp	0.144	−0.031	0.323
	Temp^2^	−0.220	−0.379	−0.070
**Abundance**				
	Intercept	0.445	0.227	0.671
	NDVI	0.422	0.225	0.623
	TPI	−0.371	−0.551	−0.181
	TWI	−0.326	−0.532	−0.129
	PRR	−0.241	−0.448	−0.034
	TWI*PRR	−0.283	−0.460	−0.106

See methods for description of parameters. Detection model parameter estimates are on the logit scale and Abundance model parameter estimates are on the log scale.

## Results

**Figure 2:**
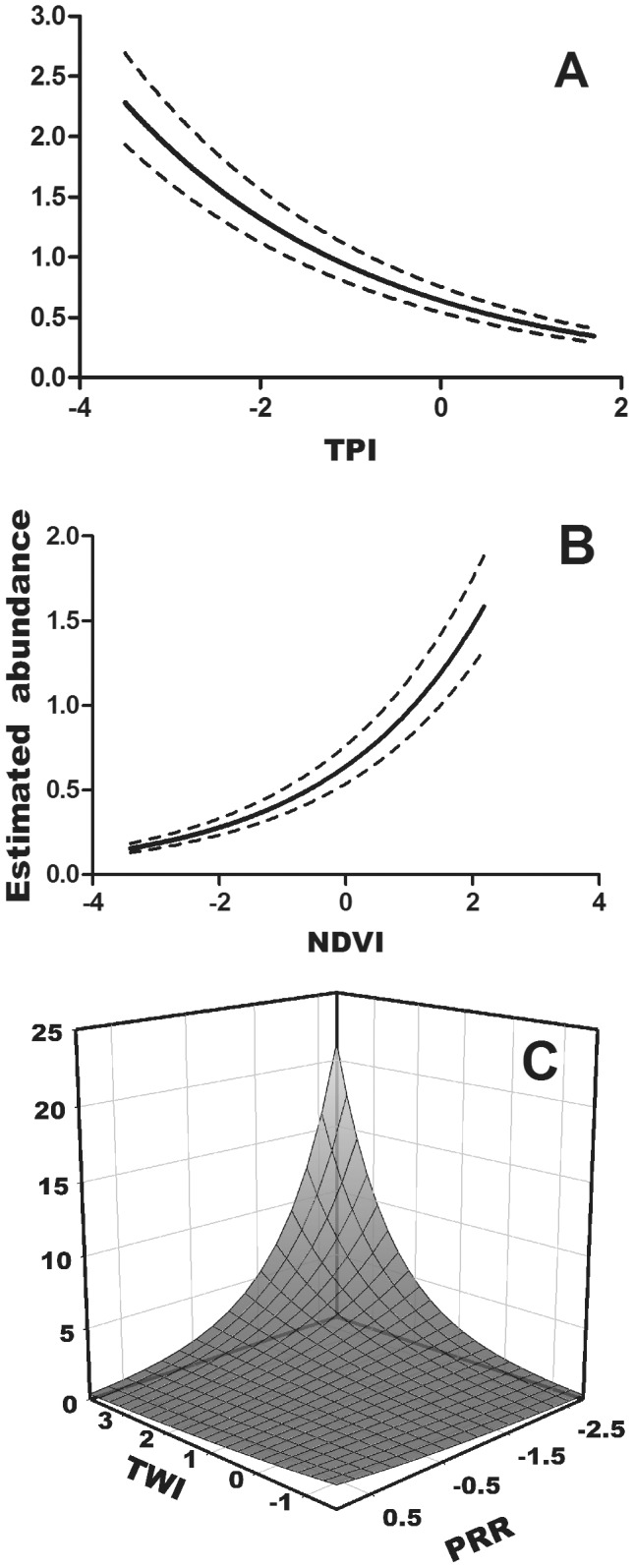
Estimated abundance relationships with (A) topographic position index; (B) canopy cover; and (C) interaction between topographic wetness and potential relative radiation. All covariates are scaled to a mean of zero. Low values of TPI indicate more ravine-like habitat and high values ridge-like habitat. Lower values of TWI, PRR, and NDVI indicate lower wetness, relative radiation, and canopy cover, respectively. Dashed lines around mean estimates of (A) and (B) represent 95% prediction intervals.

Over 7 survey periods we observed 487 salamanders at 88 of 135 surveyed plots. We found that a binomial mixture model with a random-effects parameterization of the detection process fit our data best (Bayesian p-value = 0.508; Chi-square discrepancy = 0.999). The average detection rate of salamanders across sites and observations was 0.164 (0.120–0.215 CRI), and was affected by survey date, time since the last soaking rainfall event, plot temperature, and the area of bark available to search (Table 1). After correcting for imperfect detection, we found that salamander abundance was best predicted by indices related to cooler temperatures and higher moisture. Predicted abundance was positively associated with higher canopy cover, ravine habitats (negative TPI), and areas on the landscape with low solar exposure and high topographic wetness (Table 1, Fig. 2A–C). The estimated abundance at each 9 m^2^ survey plot ranged from 0.0178–7.869 (mean = 1.968±0.687 SD).

**Figure 3:**
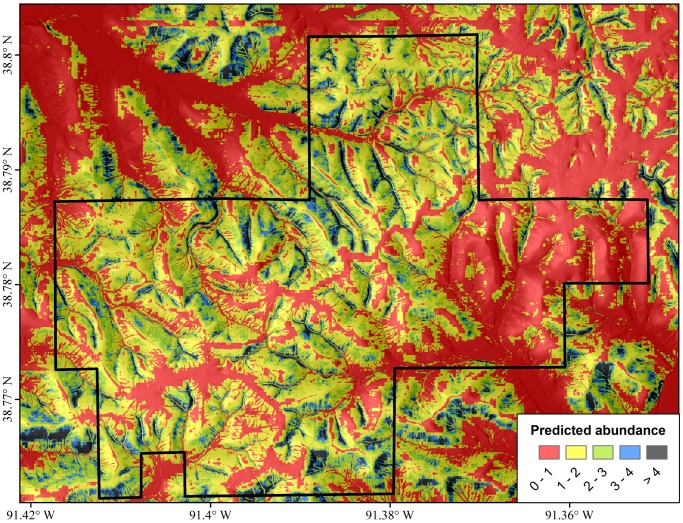
Spatial estimation of abundance across the landscape at Daniel Boone Conservation Area. Each pixel represents 9 m^2^.

**Figure 4:**
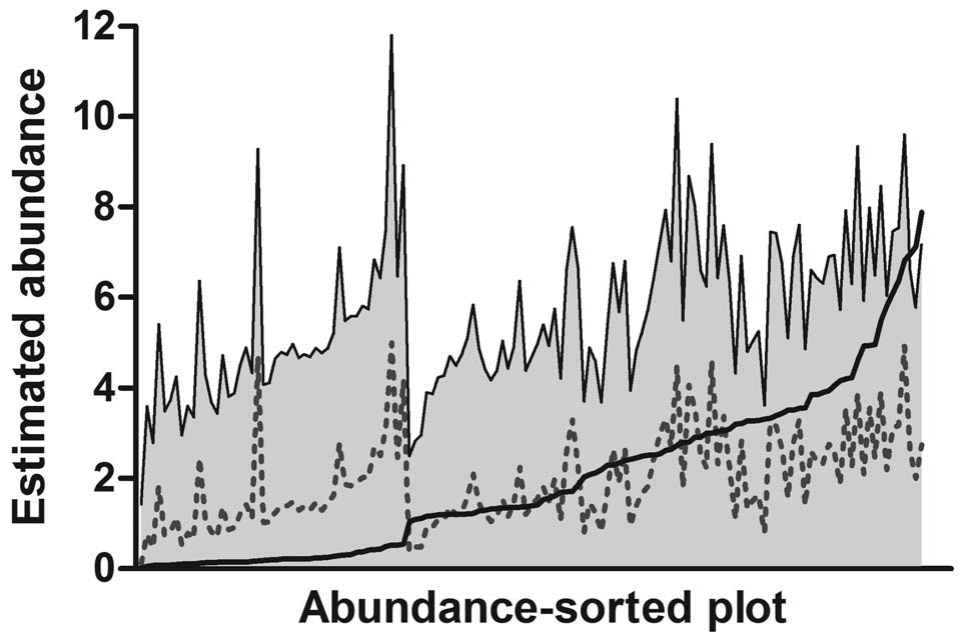
Results of the leave-one-out cross-validation of binomial-mixture model. The solid line indicates the abundance estimate for each plot from the full model, while the gray dashed line indicates the abundance estimate for each plot when observation data were omitted for that site. The gray shading indicates the 95% Bayesian credible interval around the leave-one-out plot estimate. Surveyed plots are arranged from lowest to highest predicted abundance along the x-axis.

Spatially, these relationships with environmental gradients resulted in *P. albagula* abundance being patchily distributed across the landscape (Fig. 3). Small areas of high abundance are seen in ravines, but these high abundance areas are frequently isolated from each other by hotter, drier ridges with very low estimated abundance. Our model had moderately precise predictive ability (Fig. 4). Specifically, the cross-validation estimates of plot abundance were on average 1.18 (±0.09 SD) different than the abundance estimates from the model run with the full observation data. Only five estimates of abundance exceeded the 95% credible intervals of the cross-validation test (error rate = 3.73%; Fig. 4). At sites with low predicted abundance, cross-validation over-predicted, and sites with high predicted abundance, cross-validation under-predicted. These results, in part, likely stem from the low detection rate and the model’s reliance on repeated observations to estimate the latent abundance parameter.

**Figure 5:**
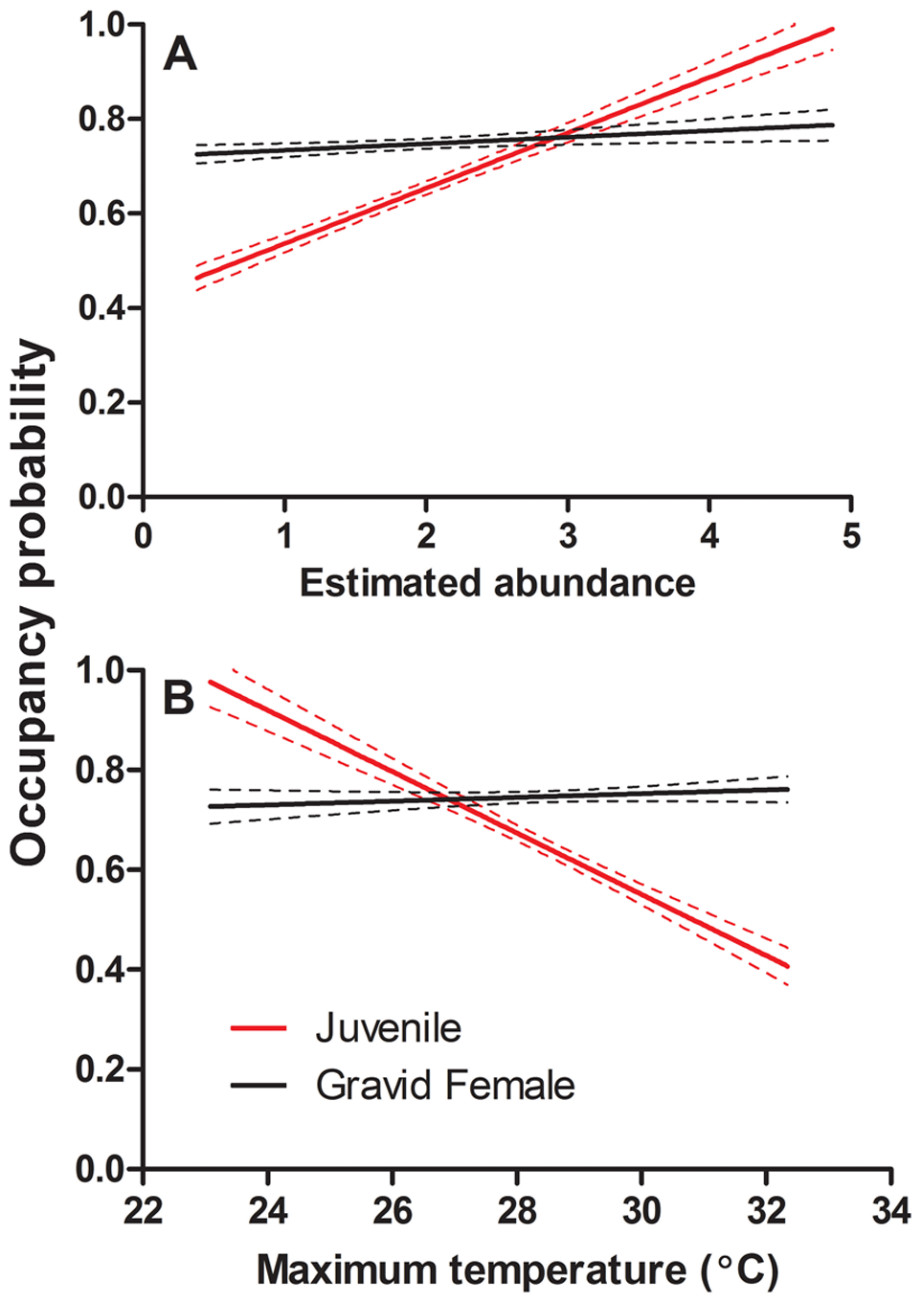
Probability that gravid female and juvenile *P. albagula* are present at occupied plots in relation to (A) the estimated abundance of the plot and (B) the maximum estimated temperature of the plot. Solid lines represent mean estimate, and dashed lines are the 95% confidence intervals.

**Figure 6:**
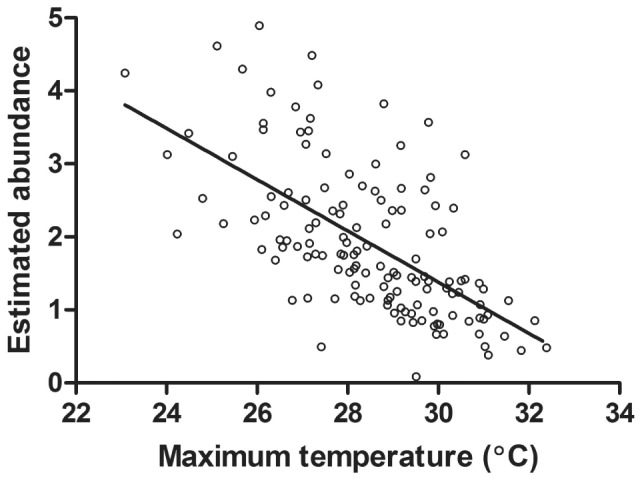
Relationship between estimated abundance and maximum surface temperature (R^2^ = 0.381, ***P*** **<0.001).**

We constructed separate multistate models for gravid females and juveniles to estimate the probability that they occur at a plot, conditional on the plot being occupied by salamanders. We found that gravid females and juveniles did not differ substantially in their respective detection probabilities (0.460±0.05 SE; 0.403±0.53 SE). In contrast, the conditional occurrence probabilities differed between the two groups. On average, females had a higher and less variable probability of occurring at an occupied plot (0.747±0.006 SE) compared to juveniles (0.645±0.013). The probability of juvenile occurrence showed a significant and positive relationship with estimated abundance (F_1,134_ = 480.50, R^2^ = 0.783, *P*<0.001; Fig. 5A), but the relationship between gravid female occurrence and estimated abundance was much weaker (F_1,134_ = 13.58, R^2^ = 0.086, *P* = 0.0003; Fig. 5A). Maximum surface temperature was not a significant predictor in the abundance model, but abundance estimates decreased significantly as surface temperature increased (F_1,134_ = 83.58, R^2^ = 0.381, *P*<0.001; Fig. 6). Because maximum temperature was not significant in the full abundance model, yet showed a strong relationship with predicted abundance, we used it as a univariate metric to assess how gravid female and juvenile occurrence probabilities differed across the landscape. The probability of juvenile salamander occurrence significantly declined as temperature increased (F_1,134_ = 190.21, R^2^ = 0.587, *P*<0.001; Fig. 5B), but there was no relationship between gravid female occurrence and maximum temperature (F_1,134_ = 1.46, R^2^ = 0.003, *P* = 0.229; Fig. 5B). There were also highly significant interactions between the juvenile and gravid female probabilities of occurrence in relation to both abundance and maximum temperature (*P*<0.001; Fig. 5A–B), suggesting a disconnect between microclimate influence on reproductive effort and successful recruitment.

## Discussion

The distribution and abundance of species along environmental gradients often results from the interplay of biotic interactions such as predation and competition with the abiotic environment [71]. The complexity of biotic interactions often makes it difficult to identify the optimal range of environmental conditions that allow a species to maximize its fitness, and studies of plethodontid salamanders have shown that species interactions are pivotal in shaping distributions along environmental gradients [31], [72]. Our study of *P. albagula* living in allopatry circumvents issues of biotic interactions with other salamanders, allowing us to clearly and directly assess distribution and abundance relationships with environmental gradients, and to make inferences concerning the population dynamics underlying the distribution of abundance across the landscape.

As we hypothesized, *P. albagula* abundance was significantly associated with environmental gradients relating to moisture and temperature. All plethodontid salamanders in the eastern United States are closely associated with mature forests [46], and abundance of plethodontid salamanders in topographically complex habitats is often greatest in ravine or cove forests [73]. In this study we found that abundance was substantially greater in areas with dense canopy cover (Fig. 2B), but within this closed canopy habitat, abundance was predicted to be more than seven times greater in ravine habitats (negative TPI) than on ridges (Fig. 2A). Canopy closure and topographic position are both critical factors influencing surface soil moisture [5], [6]. Additionally, there was a significant interactive effect of topographic wetness and solar exposure wherein areas with high moisture and low solar exposure were predicted to support the most salamanders (Fig. 2C). Moisture is well understood to be critical to surface activity and foraging [74], [75], [76], but the distribution of moist microhabitats across a topographically complex landscape and the effect on the spatial distribution of abundance has not been previously assessed in plethodontid salamanders.

The combination of environmental covariates and their relationship with salamander abundance across the landscape provides strong evidence that physiology is the underlying mechanism constraining abundance. Nonetheless, we cannot discount the potential effects that varying temperature and humidity has on prey availability, which could act as an indirect mechanism affecting salamander abundance through resource limitation [77]. Temperature and moisture both play critical roles in the physiology and ecology of terrestrial plethodontid salamanders [78], [79]. To facilitate cutaneous respiration, the skin must remain moist and permeable [80], concurrently increasing the susceptibility to water loss [81]. Further, the diffusion of gases across the skin surface, and assimilation efficiencies of nutrients, increases at cooler temperatures [78], [82]. Although the links among physiology, temperature-moisture gradients, and population growth are apparent [78], their implications for the spatial distribution of abundance and population dynamics are largely unknown. Using a mechanistic niche model, [31] projected energy budgets across a montane landscape, demonstrating that temperature and water loss were significant factors relating to surface activity and subsequent energy intake. Our results provide confirmation of this critical linkage between physiology and species abundance.

By projecting our abundance model across the study landscape (Fig. 3), we determined that the spatial pattern of abundance was not uniform. While a large proportion of the landscape is predicted to have one or more salamanders, areas of high abundance (>4) are patchily distributed among sheltered ravines. Hot, dry ridges as well as valleys with flat, floodplain-like characteristics are predicted to be largely uninhabited. Despite the negative effects that temperature can have on water loss, foraging activity, and metabolic rates, our spatial model of maximum surface temperature was not a significant predictor of abundance. We speculate that this lack of predictability occurred because the temperature surface was built from a linear model incorporating many of the same spatial attributes used to model abundance (e.g., NDVI, TWI, TPI, PRR, and distance to stream) [4]. As such, the maximum temperature surface did not account for substantial variation in abundance not already accounted for by the independently modeled parameters. Nonetheless, maximum temperature was strongly correlated with overall predicted abundance (Fig. 6).

Our final question in this study concerned the effects of environmental gradients on population dynamics. Salamander abundance is clearly not uniformly distributed across the landscape (Fig. 3), and we further demonstrated additional spatial variation in distribution of life stages through our multistate modeling. Specifically, the mean probability of a gravid female occupying a plot suitable for *P. albagula* was 0.747. There was a very weak relationship with predicted abundance (Fig. 5A) and no relationship with maximum temperature (Fig. 5B). In contrast, the probability of juvenile *P. albagula* occupancy significantly increased in relation to both predicted abundance and maximum temperature (Fig. 5A–B). These interacting relationships highlight a disconnect between reproductive effort (gravid females) and realized recruitment (juveniles) in relation to microclimate. Adult females appear to be uniformly distributed among occupied plots that range in maximum temperature from 23–33°C, but only in cooler plots (<27°C) do juveniles have an equivalent or greater probability of occurring. Foraging time for salamanders is highly dependent on local temperature and moisture conditions, with water loss dictating foraging duration [79]. Smaller, juvenile salamanders that have significantly greater surface area relative to their mass will experience greater rates of water loss, further curtailing their foraging time relative to adults [78], and restricting them to cool, moist areas.

Critical to the accuracy of our abundance estimates was the correction for imperfect detection. Plethodontid salamanders exhibit highly variable surface activity in both space and time [83], and this was true in our study. The issue of detection has contributed to the difficulty in estimating local abundance of plethodontid salamanders as well as determining relationships of abundance with local or regional landscape features [84], [85]. In our study, we had a low average detection rate (0.164; 0.120–0.215 CRI) that depended upon rainfall and temperature. After accounting for unexplained observation error in our detection model, our abundance model produced biologically realistic estimates of abundance that varied meaningfully with environmental gradients. Large woodland salamanders such as *P. albagula* have been estimated to occur at densities ranging from 0.418–0.844 m^−2^ (adults and juveniles combined) [48]; the mean density in our study was 0.219 m^−2^ with a maximum of 0.874 m^−2^. The cross-validation test of our model highlights the affect that low detection can have on abundance estimation, and the importance of correcting for these biases through repeated observations (Fig. 4). Nonetheless, the cross-validation demonstrates that our model captures the essence of the system.

*Plethodon albagula*, especially juveniles, are more frequently encountered and more abundant in cooler, moister microclimates on the landscape. These findings are corroborated with our predicted abundance model, the demographic differences observed in the multistate modeling, and with plethodontid physiology. Our study did not determine whether areas of low predicted abundance, which are more likely to be occupied by gravid females than juveniles (Fig. 5A), exist as viable populations, harbor animals dispersing between high abundance patches, or represent sink populations being supported by high-abundance ravine populations. Studies on plethodontid salamanders have identified significant genetic differentiation over small spatial scales similar to what we studied [86], [87], thus suggesting that dispersal is generally very limited [27]. No studies have yet incorporated natural habitat variation as a factor affecting salamander movement and spatial structuring of populations. A clearer understanding of *P. albagula* movement ecology is required to gain further insight into the mechanisms underlying patterns of salamander abundance across the landscape. Although we describe a general phenomenon concerning the abundance-habitat relationships of terrestrial salamanders, few studies have sought to rigorously describe the abundance-habitat relationships of terrestrial salamanders. Despite this trend, we feel that abundance-habitat relationships provide the necessary lens for understanding other critical processes such as population dynamics, local adaptation, movement and dispersal, ecophysiology, climate change, and conservation biology. Our study elucidates these foundational relationships and demonstrates a strong connection between fine-scale environmental gradients, species abundance, and population dynamics.

## Supporting Information

Table S1 Complete list of parameters used in hierarchical binomial mixture model. Only parameters whose 95% credible interval did not overlap zero were retained in the final model. These parameters are listed in Table 1, and also italicized and bolded below. See methods section for details on parameter selection.
(DOCX)
