# Supplementary material for: Fine-Scale Habitat Associations of a Terrestrial Salamander: The Role of Environmental Gradients and Implications for Population Dynamics
Source: PLoS One. 2013 May 6;8(5):e62184. doi: 10.1371/journal.pone.0062184 (PMC3646024; doi:10.1371/journal.pone.0062184)
Supplement: Table S1 — Complete list of parameters used in hierarchical binomial mixture model. Only parameters whose 95% credible interval did not overlap zero were retained in the final model. These parameters are listed in Table 1, and also italicized and bolded below. See methods section for details on parameter selection. (DOCX) [file pone.0062184.s001.docx]

| **Model** | **Parameter** |
| --- | --- |
| **Detection** | |
|  | ***Date*** |
|  | Date^2^ |
|  | 5Day.Precip |
|  | ***Soak.Rain*** |
|  | ***Soak.Rain^2^*** |
|  | Total.Rain |
|  | Total.Rain^2^ |
|  | ***Plot.Temp*** |
|  | ***Plot.Temp^2^*** |
|  | ***Bark*** |
|  | Rock |
|  | Total.Cover |
|  |  |
| **Abundance** | |
|  | Max.Temp |
|  | Max.Temp^2^ |
|  | ***NDVI*** |
|  | Slope |
|  | Eastness |
|  | Westness |
|  | Stream.Distance |
|  | Curvature |
|  | ***TPI*** |
|  | TPI2 |
|  | TWI |
|  | TWI2 |
|  | ***PRR*** |
|  | NDVI*PRR |
|  | TPI*PRR |
|  | ***TWI*PRR*** |
